# Short- and long-term costs of sinus balloon sinuplasty and middle meatal antrostomy

**DOI:** 10.1007/s00405-024-09108-8

**Published:** 2024-12-26

**Authors:** Johanna Luukkanen, Teemu Harju, Markus Rautiainen, Ilkka Kivekäs

**Affiliations:** 1 Department of Otorhinolaryngology, Head and Neck Surgery, Tampere University Hospital, PO Box 272, FI-33101 Tampere, Finland; 2 Faculty of Medicine and Health Technology, Tampere University, Tampere, Finland

**Keywords:** Balloon sinuplasty, Endoscopic sinus surgery, Middle meatal antrostomy, Chronic rhinosinusitis, Costs

## Abstract

**Purpose:**

To evaluate and compare hospital related costs, postoperative costs, and the long-term costs of maxillary balloon sinuplasty (BSP) and middle meatal antrostomy (MMA) in patients with chronic rhinosinusitis.

**Methods:**

Data were collected from patient registers on 88 patients treated with BSP and 240 patients treated with MMA between 2011 and 2017. Information was also gathered on the related costs of surgery, material, postoperative ward care, and any extra patient visits that took place within one year following the operation. The costs of sick leave were estimated based on the number of sick leave days.

**Results:**

The total costs of BSP (mean 3382 €, 95% CI 3157–3607) within one year after the operation were lower than those of MMA (mean 4546 €, 95% CI 4297–4796) (*p* < 0.001). However, the hospital related 24 h perioperative costs were higher in BSP (mean 1853 €, 95% CI 1781–1925) than in MMA (mean 1581 €, 95% CI 1515–1647) (*p* < 0.001). The number of sick leave days after BSP (mean 4 days) was half that of MMA (8 days) (*p* < 0.001), leading to an increase in sick leave costs after MMA. The duration of the BSP operation was significantly shorter than that of MMA.

**Conclusion:**

BSP of the maxillary sinus ostium appears to be less expensive than MMA when the total costs are calculated one year after the operation. This information should be considered in the preoperative visit.

**Level of evidence:**

3.

## Introduction

Balloon sinuplasty (BSP) and endoscopic sinus surgery (ESS) are both widely used surgical intervention techniques for the treatment of medical refractory chronic rhinosinusitis (CRS) [1]. Previous studies have found no difference between BSP and ESS regarding the effect on the quality of life of patients or in symptom reduction [2–7].

A thorough cost analysis of a medical intervention should consider both the costs of the health care service provided and any additional expenses. These additional expenses include the costs of the time expended by the patient for the intervention, the costs associated with the provision of care, the economic costs borne by employers, other employees, and society, and the costs associated with illness and the non-health impacts of the intervention [8]. In practice, however, it is difficult to measure some of these costs.

In 2008, Friedman et al. [9] reported that the hospital related costs during the 24 h perioperative period of BSP and ESS for primary procedures were similar, but the cost for revision surgery was lower using BSP. Moreover, the length of postoperative sick leave [10] and recovery time after the operation [3, 7] is shorter after BSP than ESS. The need for revision surgery also increases resource use and costs, but evidence regarding the difference in revision surgery rates following BSP and ESS is contradictory [3, 4, 11]. In addition, the disposable balloon instrumentation used in BSP increases costs compared to ESS. Despite prior research, however, the difference between the long-term and postoperative costs of BSP and ESS remains undetermined.

The purpose of this study was to compare hospital related costs, postoperative costs, and long-term costs related to a group of patients with CRS who underwent BSP of the maxillary sinuses (the BSP group) and a comparable group of patients who underwent middle meatal antrostomy (MMA) ESS (the MMA group).

## Materials and methods

### Patient selection

Data were collected retrospectively from the patient register of Pirkanmaa Hospital District using Nordic Classification of Surgical Procedures’ (NCSP) codes DMB20 (Endoscopic middle meatal antrostomy) and ZXB87 (Balloon dilation of nasal sinuses). Patients included in the study underwent either an MMA or a BSP operation of the maxillary sinuses due to mild chronic sinusitis primarily confined to the maxillary sinuses at Tampere University Hospital between January 2011 and December 2017. Whether MMA or BSP was chosen depended on the surgeon’s individual preference and expectations. The balloon catheter instrumentation was manufactured by Acclarent Inc. (a member of Johnson & Johnson Company, New Brunswick, NJ, USA). This study was accepted by the head of the Institutional board with the ID number R22566.

Exclusion criteria: odontogenic sinusitis, maxillary sinus cysts, need for polypectomy, or any other surgical procedure performed outside the maxillary sinus ostium in the target operation. In addition, 27 patients met the inclusion criteria but were excluded because they participated in a prospective study that had multiple study visits and other confounders. Moreover, 1 patient from the BSP group and 2 patients from the MMA group were excluded from the study because they either moved to a different hospital district within a year or were initially followed up at another hospital, resulting in missing follow-up data.

A total of 328 patients were included in the final analysis. Of these, 88 patients (27%) underwent BSP and 240 (73%) underwent MMA.

### Patient characteristics and operation data

Data collection on patient characteristics included age, gender, tobacco use, allergies, underlying diseases, and indication for the operation. Information collected on the operations involved the surgery technique, material costs, number of personnel involved, length of the operation, time spent in an operating room (OR), time spent in a post-anesthesia care unit (PACU), overnight stay, form of anesthesia, and the cost of the operation. The operation started when the local anesthetic was applied in the nasal cavity and ended when the surgical procedure ended. Local anesthetic was also applied in patients who had general anesthesia. No postoperative antibiotics were routinely administered after the operation. All the operations were performed in a hospital OR.

The length of postoperative sick leave was determined as a continuous sick leave period starting from the operation. Possible subsequent additions to the number of sick leave days were only included in the analysis when no gap existed in the middle of the sick leave period.

The BSP and MMA groups were identical in most baseline characteristics and comorbidities (Table 1).

**Table 1:** Baseline demographics and patient characteristics by operation technique

Characteristics	BSP *N* = 88	MMA *N* = 240	*p*-value
	Mean ± SD or n (%)	Mean ± SD or n (%)	
Age, y	39.6 ± 16.9	41.2 ± 15.4	0.53^a^
Gender			0.12^b^
Female	69 (78%)	167 (70%)	
Male	19 (22%)	73 (30%)	
Smoker	13 (19%)	35 (17%)	0.74^b^
NA	18 (20%)	32 (13%)	
Pollen allergy	27 (37%)	68 (30%)	0.30^b^
NA	14 (16%)	14 (6%)	
Underlying medical condition	51 (59%)	153 (64%)	0.41^b^
NA	2 (2%)	2 (1%)	

*BSP* balloon sinuplasty; *MMA* middle meatal antrostomy; *SD* standard deviation; *NA* not available

^a^The *p*-value from Mann-Whitney *U* test

^b^The *p*-value from Pearson chi-square test

### Postoperative events

Usually, one planned control visit occurred approximately one month after the MMA or BSP operation. All other visits occurring within one year after the operation were designated as extra visits, and information on the costs related to these extra visits was collected. If a complication led to the hospitalization of a patient, this was also considered as an extra visit and the costs were analyzed.

Further information on subsequent sinus operations after the primary BSP or MMA operation was gathered at 1 and 4 years after the original surgery.

### Assessment of complications

Minor postoperative bleeding, pain, and blocking of the nose were considered normal. However, if bleeding or pain led to extra health care visits before the planned postoperative appointment, they were considered as complications. Infections were judged to be complications if they occurred before the planned postoperative visit and required treatment with antibiotics. The assessment of adhesions was made during the planned postoperative visit mostly with nasal endoscopy and compared with the preoperative situation.

### Cost assessment

The costs of the surgery, immediate postoperative ward care, and any extra visits within the first postoperative year were gathered from the patient registers. The Tampere University Hospital is a non-profit public hospital. The costs in our hospital are based on a detailed cost accounting system that incorporates several factors. These include the diagnosis, the performed operation, the duration of the operation and PACU care, the number of personnel in the OR, the material costs, the costs of the ward care, the care intensity of the patient, and anesthesia. This comprehensive approach ensures that all relevant costs are accounted for truthfully.

The cost of sick leave was calculated using 350 € as the cost of one sick leave day. This figure is an estimate of the costs of one sick leave day for an employer in Finland provided by the Confederation of Finnish Industries [12].

The costs of revision surgeries were not assessed in this analysis, and their associated expenses are consequently excluded. Undergoing a revision surgery would substantially escalate overall surgery costs due to the necessity of multiple surgical interventions.

### Statistical analysis

Statistical Analysis was performed using SPSS (IBM SPSS Statistics for Windows, Version 26; IBM Corp., Armonk NY). Continuous variables were analyzed using two-sided independent samples testing, and categorical variables were analyzed using chi-square (χ^2^) tests. If Pearson chi-squared test’s conditions were unfulfilled, Fisher exact test was used. Descriptive statistics were performed to determine general cohort demographics and characteristics.

## Results

### Costs

The costs of the operation (including materials, surgery, PACU treatment, and potential ward care after surgery) were significantly greater (17%) in BSP (mean 1853 €) than in MMA (mean 1581 €). The mean material costs of BSP were 711 € greater than the mean material costs of MMA (*p* < 0.001) (Table 2).

**Table 2:** Subdivision of costs in BSP and MMA

	BSP	MMA	*p*-value^a^
	Mean [95%CI]	Mean [95%CI]	
Material costs, €	730 [645,816]	19 [13,25]	< 0.001
Operation total charge, €	1853 [1781,1925]	1581 [1515,1647]	< 0.001
Sick leave costs, €	1456 [1261,1650]	2771 [2553,2989]	< 0.001
Postoperative 1-year extra visit costs, €	73 [26,120]	195 [115,274]	0.13
Total costs in 1 year, €	3382 [3157,3607]	4546 [4297,4796]	< 0.001

*BSP* balloon sinuplasty; *MMA* middle meatal antrostomy; *CI* confidence interval

^a^The *p*-value from Mann-Whitney *U* test

The length of sick leave was almost half shorter (47%) in BSP than in MMA (*p* < 0.001) (Table 3), leading to lower sick leave costs associated with BSP (47%) as well. The mean sick leave costs were 1456 € in BSP and 2771 € in MMA (Table 2). No difference was detected in the costs of any additional visits occurring within the first postoperative year (Table 2).

**Table 3:** Variables that affect the operation costs of BSP and MMA

	BSP	MMA	*p*-value
	Mean ± SD or n (%)	Mean ± SD or n (%)	
*Operation statistics*			
Time spent in OR, min	61 ± 13	71 ± 21	< 0.001^a^
Duration of operation, min	35 ± 13	47 ± 20	< 0.001^a^
Duration of PACU care, min	60 ± 48	73 ± 55	0.054^a^
Combined duration (OR + PACU), min	121 ± 52	144 ± 60	< 0.001^a^
Number of OR personnel	5 ± 0.4	5 ± 0.5	0.11^a^
Patients needing overnight stay	2 (2%)	20 (8%)	0.052^b^
Local anesthesia	46 (52%)	147 (61%)	0.14^b^
General anesthesia	42 (48%)	93 (39%)	0.14^b^
*Sick leave*			
Total duration of sick leave, d	4.2 ± 2.6	7.9 ± 4.9	< 0.001^a^
Original duration of sick leave, d	4.1 ± 2.5	7.6 ± 4.5	< 0.001^a^
Patients needing extra sick leave	2 (2%)	12 (5%)	0.37^c^
*New sinus surgery*			
In 1 year postoperatively	2 (2%)	5 (2%)	1.00^c^
In 4 years postoperatively	6 (7%)	15 (6%)	0.85^b^
*Additional visits in 1 year*			
Patients having 1 or more extra visits	14 (16%)	55 (23%)	0.17^b^
Number of extra visits in 1 year	0.2 ± 0.6	0.5 ± 1.3	0.15^a^

*BSP* balloon sinuplasty; *MMA* middle meatal antrostomy; *SD* standard deviation

^a^The *p*-value from Mann-Whitney *U* test

^b^The *p*-value from Pearson chi-square test

^c^The *p*-value from Fisher exact test

When the costs of the operation, sick leave, and any additional visits within the first postoperative year were calculated, the total costs of BSP (mean 3382 €) were cheaper (26%) than MMA (mean 4546 €) (*p* < 0.001) (Table 2).

### Factors affecting costs

The mean duration of the operation and the mean overall time spent in the OR were both significantly shorter in BSP than in MMA (Table 3). The difference in PACU care duration between the groups was not significant. Furthermore, most patients had no extra visits in addition to the normal postoperative appointment. There was no difference in the number of postoperative visits per patient, the re-operation rate, or the number of OR personnel (Table 3). All revision procedures were performed due to the persistence or recurrence of rhinosinusitis symptoms after surgery.

In total, two (2%) patients from the BSP group and 20 (8%) patients from the MMA group spent the first postoperative night in hospital, but the difference was statistically insignificant (*p* = 0.052) (Table 3). However, an overnight stay in hospital raised the total cost of the operation (mean 2117 €, 95% CI 1857–2377) compared to situations where the patient was discharged on the day of the operation (mean 1620 €, 95% CI 1568–1673) (*p* = 0.003). Of the 22 patients who were hospitalized for the first postoperative night, 6 (27%) had the operation under local anesthesia and the remaining 16 (73%) under general anesthesia (*p* = 0.002) (not shown in tables). However, no difference existed in the form of anesthesia between the BSP and MMA groups (Table 3).

### Complications

No difference between the BSP and the MMA groups emerged regarding postoperative bleeding, infections, or pain leading to extra health care visits. The number of postoperative adhesions in the nasal cavity and the middle meatus was, however, significantly higher after MMA. (Table 4).

**Table 4:** Complications occurring after BSP and MMA operations

	BSP	MMA	*p*-value
	n (%)	n (%)	
Unusual bleeding	1 (1%)	6 (3%)	0.68^b^
Postoperative infection	11 (14%)	37 (16%)	0.67^a^
Unusual pain	2 (3%)	5 (2%)	1.00^b^
Adhesions	5 (6%)	57 (24%)	< 0.001^a^

*BSP* balloon sinuplasty; *MMA* middle meatal antrostomy

^a^The *p*-value from Pearson chi-square test

^b^The *p*-value from Fisher exact test

## Discussion

This study has shown that when the additional costs outside the health care service costs are acknowledged, BSP is cheaper than MMA. The difference in mean total cost between BSP and MMA in the one-year follow-up period was 1164 €. This is new information. A prior study comparing the costs of BSP and ESS covered only the costs of the 24 h perioperative period [9]. In addition to maxillary procedures, the study in question also examined operations to the frontal, ethmoid, and sphenoid sinuses. In that study, the costs of BSP and ESS for primary procedures were similar, but the cost for revision surgery was lower using BSP. Dissimilarities in disease type and observation time explain the differing results provided by that study.

As BSP instrumentation has commonly been thought to be expensive, it was important to further evaluate the total costs of both BSP and MMA. Initially, MMA was cheaper when only the health care service costs of the operation were compared. Indeed, the material costs of BSP were 711 € more expensive and included the cost of balloon device instrumentation. However, the mean cost of the operation during the perioperative period was only 272 € (17%) greater in BSP. This suggests that MMA might associate with other factors, unrelated to the price of instrumentation, which increase the costs of the operation.

The costs of an operation often comprise the services and materials used, the length of time, the intensity of the care given, and the number of personnel. The surgery costs at our hospital were calculated using a comprehensive cost calculation system, where the services and materials used, the intensity of the given care, the time, and the diagnosis were all considered. The aim of this cost calculation was to create the most reliable objective estimate of the actual costs. In this present study, discrepancies between surgical techniques were detected in the operation durations and the time spent in the OR. In the BSP procedure, the mean duration of the operation itself was 12 min shorter, and the total mean time spent in the OR was 10 min shorter compared to the MMA procedure. These factors explain, at least partly, why the difference in the operation costs was not as large as the difference in the material costs.

Koskinen et al. [10] detected no significant differences in the duration of BSP and MMA (BSP mean 35 min vs. MMA mean 29 min). However, in their study, all MMA patients underwent surgery in the OR of a public hospital, whereas all the BSP patients underwent surgery in two private clinics. Furthermore, several BSP procedures were performed in an in-office setting. In practice, these differences possibly led to the differing results between our study and the study in question.

The instrumentation used, anesthesia, and sedation all influence the duration of an operation and OR time. BSP was a rather new procedure at the beginning of the data collection period, and this is probably why general anesthesia was quite often used (48%). If the rate of general anesthesia had been lower, it could have possibly lowered the cost of BSP even more. Moreover, as BSP can also be performed as an in-office procedure [3, 4, 13], costs could potentially be lowered even further, as shown by Prickett et al. [14]. In that study, the charges for rhinologic procedures performed in the office were compared with those for similar procedures performed in the OR, and the mean total charges for office-based procedures were lower than for OR procedures (2737 $ US vs. 7330 $ US, *p* < 0.001).

Patients who were hospitalized for the first postoperative night were significantly more likely to have had general anesthesia than local anesthesia. Although no significant difference was detected between the surgery techniques in the overnight stay rate (*p* = 0.052), 8% in the MMA group stayed the night but only 2% in the BSP group. This 6% difference might have raised the costs of MMA even further, as the mean operation total cost was 497 € higher when the patient stayed the first postoperative night in hospital.

The recovery time has already been shown to be shorter with BSP than ESS, both in anterior ± frontal procedures [7] (2.2 days vs. 5 days) and in anterior procedures [3, 4] (1.6 days vs. 4.8 days; *p* = 0.002) and (1.7 vs. 5.0 days; *p* < 0.0001). Koskinen et al. [10] compared the length of sick leave after BSP and MMA and found that the sick leave was shorter after BSP (mean 4 days) than MMA (mean 14 days) (*p* < 0.001). A similar trend of shorter sick leave after BSP (mean 4.2 days) was also seen in our study, although the mean sick leave after MMA was 7.9 days. This difference is distinct and arouses the need to reflect on how sick leave length is determined. In this study, the surgeon based the length of sick leave on an educated guess, practical experience, and institutional customs. Therefore, further research and practice guidelines are needed to cut the number of expensive unnecessary sick leave days.

The costs of sick leave were almost half cheaper in BSP than in MMA because the sick leave was half shorter. Indeed, the costs of sick leave proved so large that they turned the total costs in favor of BSP instead of MMA. All possible recovery time costs, outside mere sick leave costs, are hard to measure. Therefore, in practice, the recovery time costs may have been even more expensive. The results of this study clearly show that recovery time costs should not be underestimated when calculating the operation costs in future.

No differences emerged in the number of patients with postoperative bleeding, infections, or pain. Prior studies have also reported that the complication rate in BSP and ESS is low and no difference in complications exist between these techniques in either maxillary procedures [10], maxillary ± ethmoid procedures [4] or maxillary ± ethmoid ± frontal recess procedures [7]. However, BSP of the maxillary sinus ostium appears to cause less adhesions than maxillary ESS [2, 10], which was also detected in this study. Nevertheless, only one patient underwent re-ESS due to adhesions in OR and two patients needed extra visits for adhesion dissection in the outpatient clinic. In the study by Koskinen et al. [10], none of the adhesions obstructed the ostiomeatal area. In the light of these findings, it seems that the adhesions are only a slight inconvenience.

When BSP and ESS are compared, prior evidence of relapse surgery is conflicting. In anterior procedures at 6 months [3] and at 12 months [4], no difference in the revision rate was found between BSP and ESS. Koskinen et al. [11], however, detected a significantly higher risk for revision surgery in maxillary BSP (17 out of 77 patients) than in MMA (6 out of 82 patients). The mean follow-up time was long: 5.3 years in the BSP group and 9.8 years in the MMA group. In the present study, no difference in the revision surgery rate was detected at 1 and 4 years. Cost data for revision surgeries were not collected, and therefore their costs could unfortunately not be analyzed in the total cost assessment. In both the BSP and MMA groups, 2% of patients required revision surgery within one year of the initial procedure. In both groups, all revision surgeries were performed using surgical methods, and BSP was not used again to dilate the sinus ostia. Thus, the costs of the revision surgeries are likely comparable between the groups, considering the surgical costs, sick leave costs, and additional postoperative costs due to complications or other factors. As a result, the costs of the revision surgeries would unlikely have significantly altered the overall cost distribution between the procedures.

### Study limitations

Due to the retrospective nature of this study, we are unable to determine the exact reasons behind the choice between the MMA and BSP approaches. It is possible that in more complex cases, endoscopic sinus surgery may have been preferred.

Owing to the retrospective design, limitations to data collection occurred due to missing information. In addition, limitations concerning sick leave and the costs of sick leave do exist. The length of sick leave was determined by the surgeon, and occasionally it might have been too long. The price of one sick leave day 350 € is an estimate given by the Confederation of Finnish Industries [12] and is only an evaluation of the costs to employers. Other costs, for example to society, are not included. As some additional expenses in addition to health care service costs are hard to measure, these costs were not examined. The prices given are in euros and represent the level of costs in Finland between 2011 and 2017. The results of the present study can, however, be generalized globally if only local sick leave cost levels and local costs for OR and PACU duration are used.

The advantage of lower costs is heavily contingent on how the overall costs are distributed and shared among different entities. Frequently, aspects such as sick leave and other medical expenses are managed by separate operators. This segregation of responsibilities can impact the overall cost structure and efficiency of the services provided. Our study aimed to assess costs from a societal perspective rather than solely from the patient’s, employer’s, or provider’s standpoint.

This study focused only on the maxillary sinuses. Further research is, therefore, needed to evaluate the costs of more extensive sinus and nasal surgery. Further assessment of the number of sick leave days after sinonasal surgery will also be required in future.

## Conclusion

When total costs one year after the operation are calculated, BSP of the maxillary sinus ostium appears to be less expensive than MMA. Even though the perioperative health care service costs of MMA are lower, the longer sick leave period after MMA results in higher recovery time expenses, making the total costs of BSP ultimately cheaper. Because prior studies have not reported a difference between BSP and MMA in the effect on a patient’s quality of life, we should use cost as a guide when choosing between these two surgical techniques.
